# pH-mediated upregulation of *AQP1* gene expression through the Spi-B transcription factor

**DOI:** 10.1186/s12867-018-0104-9

**Published:** 2018-03-20

**Authors:** Yihui Zhai, Hong Xu, Qian Shen, Franz Schaefer, Claus P. Schmitt, Jing Chen, Haimei Liu, Jialu Liu, Jiaojiao Liu

**Affiliations:** 1 Department of Nephrology and Rheumatology, Children’s Hospital of Fudan University, No. 399 Wanyuan Road, Shanghai, 201102 China; 2 Shanghai Kidney Development and Pediatric Kidney Disease Research Center, Shanghai, China; 3 Division of Pediatric Nephrology, Center for Pediatric and Adolescent Medicine, University of Heidelberg, Heidelberg, Germany

**Keywords:** Aquaporin 1, pH, Promoter, Transcription, Peritoneal dialysis fluids

## Abstract

**Background:**

Bicarbonate-based peritoneal dialysis (PD) fluids enhance the migratory capacity and damage-repair ability of human peritoneal mesothelial cells by upregulating AQP1. However, little is known about the underlying molecular mechanisms.

**Results:**

Here we used HEK-293T cells to investigate the effect of pH on *AQP1* gene transcription levels. We found that *AQP1* mRNA levels increases with pH. Transfection of HEK-293T cells with luciferase reporter vectors containing different regions of the *AQP1* promoter identified an upstream region in the *AQP1* gene between − 2200 and – 2300 bp as an enhancer required for pH-mediated regulation of *AQP1* expression. Site-directed mutagenesis of this specific promoter region revealed a critical region between − 2257 and − 2251 bp, and gene knock-down experiments and ChIP assays suggested that the Spi-B transcription factor SPIB is involved in pH-mediated regulation of AQP1 expression.

**Conclusions:**

We identified an upstream region in the *AQP1* gene and the transcription factor SPIB that are critically involved in pH-mediated regulation of AQP1 expression. These findings provide the basis for further studies on the pH- and buffer-dependent effects of PD fluids on peritoneal membrane integrity and function.

**Electronic supplementary material:**

The online version of this article (10.1186/s12867-018-0104-9) contains supplementary material, which is available to authorized users.

## Background

End-stage renal disease (ESRD) has a poor prognosis and affects human populations around the world [1]. Peritoneal dialysis (PD) is a well-established renal replacement therapy that takes advantage of the semi-permeability of the peritoneal membrane, to remove toxins and excess water from the circulatory system. Maintenance of the morphological and functional integrity of the peritoneum is essential for long-term PD patients. However, conventional acidic PD solutions cause peritoneal cell damage, cell shedding [2–4], peritoneal fibrosis and progressive vascularization [5–8], leading to ultrafiltration loss and eventually PD failure [9].

Novel PD solutions that separate glucose from the buffer at a very low pH contain fewer glucose degradation products (GDPs) and have a neutral to physiological pH after mixing. Several studies suggest that these solutions can reduce peritoneal cell damage and preserve the peritoneal membrane host defence and transport functions. The solutions contain lactate, bicarbonate or a mixture of both buffers. A prospective paediatric trial that compared a pure bicarbonate buffer to a purely lactate-based low GDP fluid demonstrated better preservation of ultrafiltration capacity over 10 months [10]. However, the underlying mechanisms remained elusive, and the respective impacts of the solutions on cellular pH are unknown. The ultrafiltration capacity of the peritoneum is closely related to peritoneal aquaporin 1 (AQP1) expression. AQP1 is expressed in peritoneal capillaries, post-capillary venules and peritoneal mesothelial cells, which are critical for maintaining local peritoneal homeostasis [11–13]. AQP1 knockout mice have approximately 50% less peritoneal ultrafiltration capacity [14, 15]. Previously, via in vitro experiments, we demonstrated pH and buffer dependent upregulation of AQP1 in human peritoneal mesothelial cells and improved AQP1-dependent cell migration. Actinomycin D-based experiments further demonstrated that the pH-mediated effects on AQP1 occurred at the transcriptional level [16]. Differences in the PD fluid buffer and pH may therefore play an important role through regulation of *AQP1* gene expression.

Thus, here, we examined the underlying molecular mechanisms of pH- and buffer-dependent AQP1 expression in HEK-293T cells, which display reliable growth and have a propensity for transfection. We extensively explored the key transcription factors and their binding sites involved in pH-mediated regulation of AQP1 expression by cloning the *AQP1* gene promoter region and various truncated forms of the promoter region.

## Materials and methods

### Plasmids and reagents

A Flag-tagged SPIB expression clone and the vector plasmid were purchased from GeneCopoeia (Guangzhou, China). Anti-IgG and anti-SPIB antibodies were purchased from Cell Signaling Technology, and anti-Flag and anti-β-actin antibodies were obtained from Sigma.

### Cell culture

The HEK-293T cell line was purchased from ATCC (American Type Culture Collection). HEK-293T cells were cultured at 37 °C with 5% CO_2_ in DMEM supplemented with 10% foetal bovine serum and transfected using Lipofectamine 2000 reagent (Invitrogen) according to the manufacturer’s instructions. The total amount of plasmid DNA used for transfection was maintained at a constant level between experimental conditions through the addition of empty vector plasmid. To investigate the regulatory effects of pH on *AQP1* gene expression, HEK-293T cells were seeded into 6-well plates. Once the cells attached to the culture surface, the regular culture medium was replaced with serum-free culture medium, and the cells were cultured in the serum-free medium for 24 h. Subsequently, the cells were treated with bicarbonate-buffered complete media in which the pH was adjusted to 6, 7 or 8 with HCl or NaOH. After treatment for 24 h, the cells were lysed.

### RNA extraction and real-time PCR analysis

Total cellular RNA was extracted using Trizol reagent (Invitrogen Corporation). In total, 500 ng of RNA was reverse transcribed into cDNA using a PrimeScript Reverse Transcription Kit (Takara Biotechnology Co., Ltd); β-actin was used as an internal reference. The sequences of the specific primers for quantitative polymerase chain reaction (qPCR) were listed in Additional file 1: Table S1. The relative expression levels of target genes were calculated using the 2^−ΔΔCT^ method.

### Western blot analysis

Cells were harvested by scraping into an SDS sample buffer. Similar protein amounts were loaded onto a 7.5% SDS-PAGE gel, separated by electrophoresis, and transferred to a nitrocellulose membrane (Bio-Rad, Hercules, CA). The membrane was blocked with TBST (0.05% Tween 20 in TBS) containing 5% skim milk and then incubated overnight with the indicated antibodies at 4 °C. The membrane was washed three times in TBST and then incubated with an HRP-conjugated secondary antibody (Pierce, Rockford, IL) (1:2000) for 2 h at room temperature. The membranes were stripped of the primary antibodies and reprobed with antibodies as necessary. The immunocomplexes were detected using enhanced chemiluminescence (Pierce, Rockford, IL, USA).

### Dual-luciferase reporter vector construction

Genomic DNA was extracted from HEK-293T cells using an AllPrep DNA/RNA Mini Kit (Qiagen). The isolated genomic DNA was used as a template for PCR amplification of the *AQP1* promoter region. According to the genomic structure information for the *AQP1* gene, the first base in the *AQP1* coding sequence was numbered + 1. The *AQP1* primer sequences were TTA CGC GTG CTA GCC CGG GCC TGC ACT TAG CAG AAG CTT CTG GCC AG (upstream primer) and GCT TAC TTA GAT CGC AGA TCA TGA AGA CAA AGA GGG TCG TGG CCA GG (downstream primer). The digested DNA fragments were ligated into a pGL3-Basic plasmid (Promega, Madison, USA). After verification via sequencing, the successfully constructed vector was named pGL3-AQP1-4K. Using pGL3-AQP1-4K, a series of luciferase reporter vectors containing various truncated forms of the *AQP1* promoter were further constructed and named pGL3-AQP1-1K, pGL3-AQP1-2K, pGL3-AQP1-3K, pGL3-AQP1-2.2K, pGL3-AQP1-2.3K, pGL3-AQP1-2.4K, pGL3-AQP1-2.6K, pGL3-AQP1-2.8K, and pGL3-AQP1-2-3K. Using pGL3-AQP1-2.3K as the template and a QuikChange Lightning Site-Directed Mutagenesis Kit, a luciferase reporter vector containing a mutated *AQP1* promoter fragment (pGL3-AQP1-2.3K mutant) was established. Additionally, the 0-1K and 2-3K regions in the *AQP1* promoter were amplified via PCR. Subsequently, overlap PCR was performed to construct the pGL3-AQP1-3Δ2K vector.

### Transfection of oligonucleotides

The EGR1, FOXL1, KLF5, NFIC, RFX5, SP1, THAP1, SPIB, and AQP1 siRNA duplexes and negative control (scrambled, has no significant sequence similarity to mouse, rat, or human gene sequences) were designed and synthesized by RiboBio (Guangzhou, China). For each cell of a six-well plate, cells were transfected with a pool of three siRNAs in a total volume of 5 μl (20 μM) and 5 μl Lipofectamine 2000.

HEK-293T cells were first seeded into 96-well plates at a density of 5 × 10^3^ cells per well 24 h before transfection. The cells were transfected with a mixture of 50 ng firefly luciferase reporter and 5 ng pRL-CMV Renilla luciferase reporter using Lipofectamine 2000. The transfected HEK-293T cells were used in the subsequent assays.

### Examination of luciferase activity

Twenty-four hours after transfection, the cells were covered with serum-free medium and cultured for an additional 24 h. Subsequently, the cells were incubated in culture medium with different pH values for 6 h. The firefly and Renilla luciferase activities of the cell lysates were measured using a Dual-Luciferase Reporter Assay System according to the manufacturer’s instructions (Promega). All activity values were normalized to those of the control vector pGL-basic 3.0 at pH 7. The results were assessed based on changes in the luciferase activity. All experiments were repeated three times.

### Chromatin immunoprecipitation (ChIP)

HEK-293T cells were first seeded into 10-cm dishes and then transfected with plasmids the next day when the cells reached approximately 40% confluence using Lipofectamine 2000. For the ChIP assay, 4 × 10^6^ cells were prepared using a SimpleChIP Enzymatic Chromatin IP kit (Cell Signaling Technology) according to the manufacturer’s instructions. Briefly, HEK-293T cells were fixed with 1% formaldehyde for 10 min at room temperature followed by glycine. Lysates were then digested with micrococcal nuclease into DNA/proteins fragments. Anti-flag or control IgG antibody was added, and the complex were co-precipitates and captured by protein G magnetic beads. After an overnight incubation at 4 °C, the complexes were purified, and the crosslinking was reversed at 65 °C. The resulting precipitated DNA samples were analyzed by PCR or real-time PCR using the primers listed in Additional file 1: Table S1.

### Statistical analysis

Each group of experiments was repeated three times or more. Each time, three replicate wells were prepared. The measurement data were expressed as the mean ± standard error of the mean (SEM). Unless otherwise noted, Student *t* test (two-tailed) and one-way analysis of variance (ANOVA) followed by the Dunnett multiple comparisons test were used to compare differences between 2 groups and more than 2 groups, respectively. Two-way ANOVA was used to analyse the luciferase activity and AQP1 mRNA levels in HEK-293T cells with different pH treatment. A P value less than 0.05 indicated that the difference was statistically significant. The above statistical analyses were conducted using GraphPad software, and the results were plotted.

## Results

### Effect of pH on AQP1 expression

**Fig. 1:**
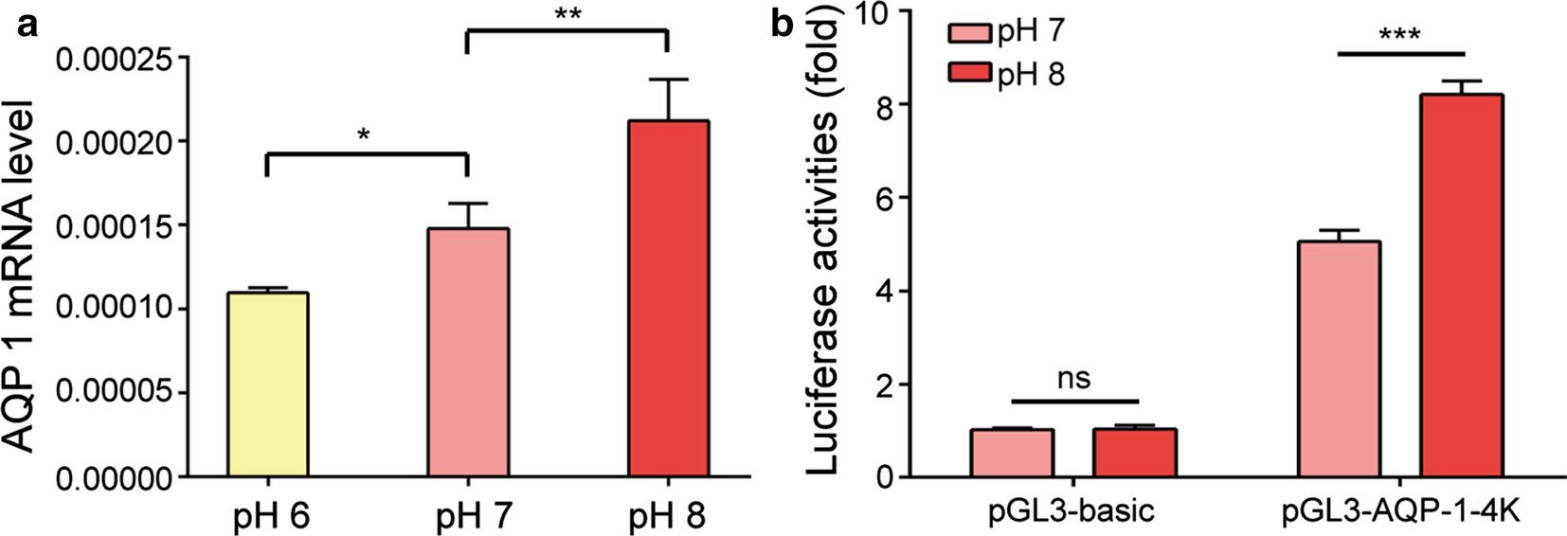
Increased pH elevates AQP1 expression. **a** AQP1 mRNA levels in HEK-293T cells with various pH treatments. **b** Luciferase activity in HEK-293T cells transfected with firefly luciferase reporter plasmids containing AQP1 upstream regions. The Renilla luciferase reporter was co-transfected with pGL3-basic or a plasmid reporter. The data represent the mean ± SEM. *ns* no statistical significance; *P < 0.05; **P < 0.01; ***P < 0.001

To investigate the regulatory effects of pH on *AQP1* gene expression, HEK-293T cells were first used as a research tool to examine whether *AQP1* mRNA levels were upregulated at certain pH levels. HEK-293T cells grown to confluence with regular culture medium were exposed to serum-free bicarbonate-buffered medium for 24 h, and the pH was adjusted to 6, 7 or 8 by the addition of HCl or NaOH. Quantitative PCR demonstrated a significant upregulation of *AQP1* expression with pH, with two-fold higher mRNA levels at pH 8 than those at pH 6 (P = 0.0009, one way ANOVA, Fig. 1a).

### Genomic structure of the AQP1 gene and construction of luciferase reporter vectors containing AQP1 promoter regions

The upstream region of the *AQP1* gene was analysed using the UCSC Genome Browser database. According to existing knowledge, we hypothesized that a 4-kb region upstream of the *AQP1* gene is the transcriptional regulatory region and contains binding sites for transcription factors. Therefore, we used HEK-293T genomic DNA as a template to clone the 4-kb upstream region of the *AQP1* gene lying between − 4000 and 0 base pairs (bp; the first base in the AQP1 coding sequence was denoted as + 1). The resulting 4000-bp fragment was ligated into a pGL3-Basic vector to establish a luciferase reporter vector containing the potential AQP1 promoter region (pGL3-AQP1-4K). The luciferase activity of pGL3-AQP1-4K and the regulatory effects of pH on luciferase activity are shown in Fig. 1b. High pH upregulated the luciferase activity of pGL3-AQP1-4K in HEK-293T cells.

### Identification of the AQP1 gene upstream promoter region

**Fig. 2:**
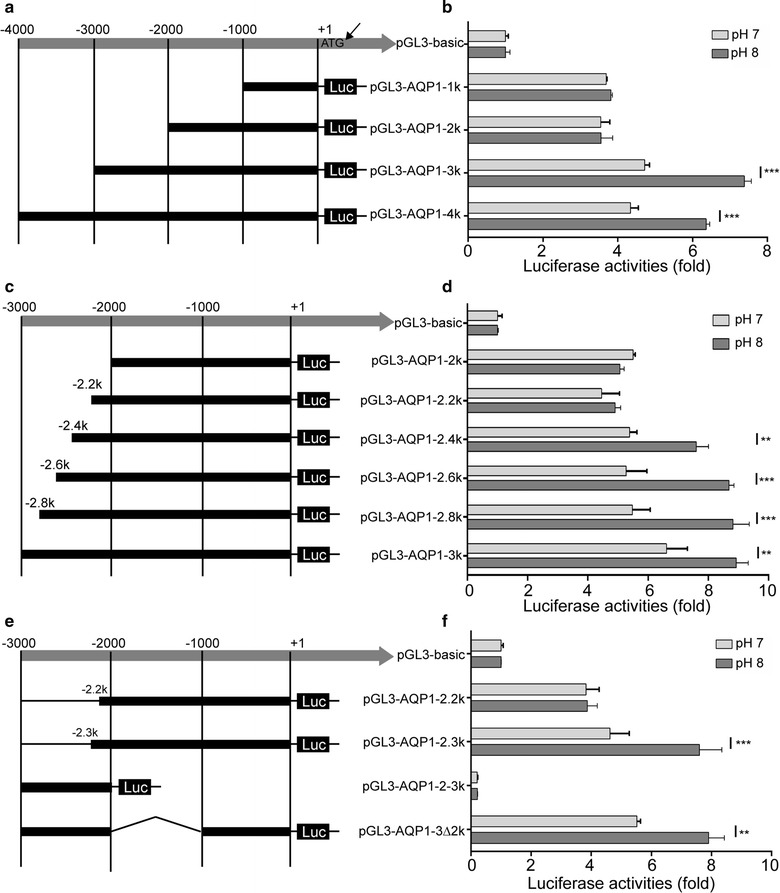
Identification of the AQP1 gene upstream promoter region. **a**, **c** and **e** Schematic representation of human AQP1 promoter reporter constructs. Fragments with various lengths of the AQP1 promoter region between − 4000 to 0 bp were cloned downstream of the firefly luciferase reporter. **b**, **d** and **f** Luciferase activity in HEK-293T cells transfected with firefly luciferase reporter plasmids containing various AQP1 upstream regions. The Renilla luciferase reporter was co-transfected with pGL3-basic or a plasmid reporter. The data represent the mean ± SEM. **P < 0.01; ***P < 0.001

We constructed a series of luciferase reporter vectors containing different regions of the potential *AQP1* promoter. HEK-293T cells were transiently transfected with the reporter plasmids, and the promoter activity of the regions was examined using a dual-luciferase reporter assay. The HEK-293T cells transfected with the luciferase reporter vector containing the *AQP1* gene upstream region between − 3000 and 0 bp displayed strong luciferase activity (Fig. 2a, b). Additionally, cells transfected with pGL3-AQP1-3K showed increased luciferase activity in response to an increase in pH. The results indicated a critical role for the 3-kb upstream region (− 3000 to 0 bp) in maintaining *AQP1* promoter activity and demonstrated that a pH-responsive region is located between − 3000 and − 2000 bp. Furthermore, transfection of HEK-293T cells with a series of luciferase reporter vectors containing various segments of the region between − 3000 and − 2000 bp demonstrated that the region between − 2300 and − 2200 bp is critical for the pH-mediated regulation of AQP1 gene expression. Moreover, the region between − 1000 and 0 bp was also required to initiate *AQP1* gene transcription (Fig. 2c–f).

### Prediction of transcription factors that interact with the enhancer region in the AQP1 gene (− 2300 to − 2200 bp)

**Fig. 3:**
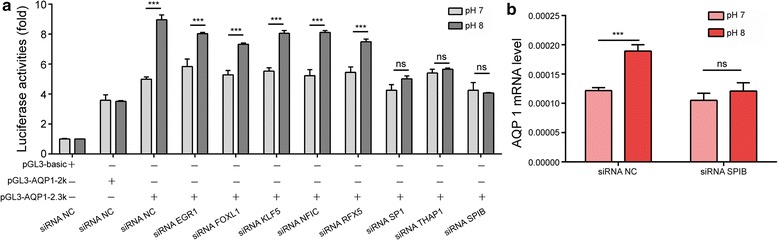
Transcription factors interacting with the enhancer region of the AQP1 gene. **a** Luciferase activity associated with the region between − 2.3k and 0 bp in AQP1 in HEK-293T cells transfected with small interfering RNAs (siRNAs) against EGR1, FOXL1, KLF5, NFIC, RFX5, SP1, THAP1, or SPIB or with a negative control (NC). **b** AQP1 mRNA levels in HEK-293T cells after SPIB knockdown and treatment at pH 7 and 8 were assessed with real-time PCR. The data represent the mean ± SEM. *ns* no statistical significance; ***P < 0.001

We predicted transcription factors that were likely to bind to the enhancer region (−2300 to − 2200 bp) in the *AQP1* gene using the JASPAR database. The predicted transcription factors were regulatory factor X5 (RFX5), early growth response 1 (EGR1), Krueppel-like factor 5 (KLF5), SP1, THAP domain-containing apoptosis-associated protein 1 (THAP1), E74-like factor 5 (ELF5), ETS homologous factor (EHF), Spi-B transcription factor (SPIB), nuclear factor I-C (NFIC), T cell leukaemia homeobox 1 (TLX1) and forkhead box L1 (FOXL1). Next, we transfected HEK-293T cells with siRNAs against the 11 transcription factors identified. At 48 h after transfection, RNA was collected and reverse transcribed into cDNA. The interference efficiency of each siRNA was determined. The ELF5, EHF and TLX1 genes were not expressed in HEK-293T cells, and the expression of the other 8 genes was significantly reduced after siRNA-mediated interference (Additional file 1: Fig S1). Furthermore, HEK-293T cells were transiently co-transfected with siRNAs against the 8 genes and the luciferase reporter vector containing the 2.3-kb region between − 2300 and 0 bp. Subsequently, the cells were exposed to different pH levels, and changes in the luciferase activity were examined. The results showed that changes in pH failed to induce corresponding changes in luciferase activity after siRNA-mediated downregulation of SP1, SPIB and THAP1 (Fig. 3a).

### SPIB is a potential transcription factor capable of influencing pH-regulated AQP1 expression

**Fig. 4:**
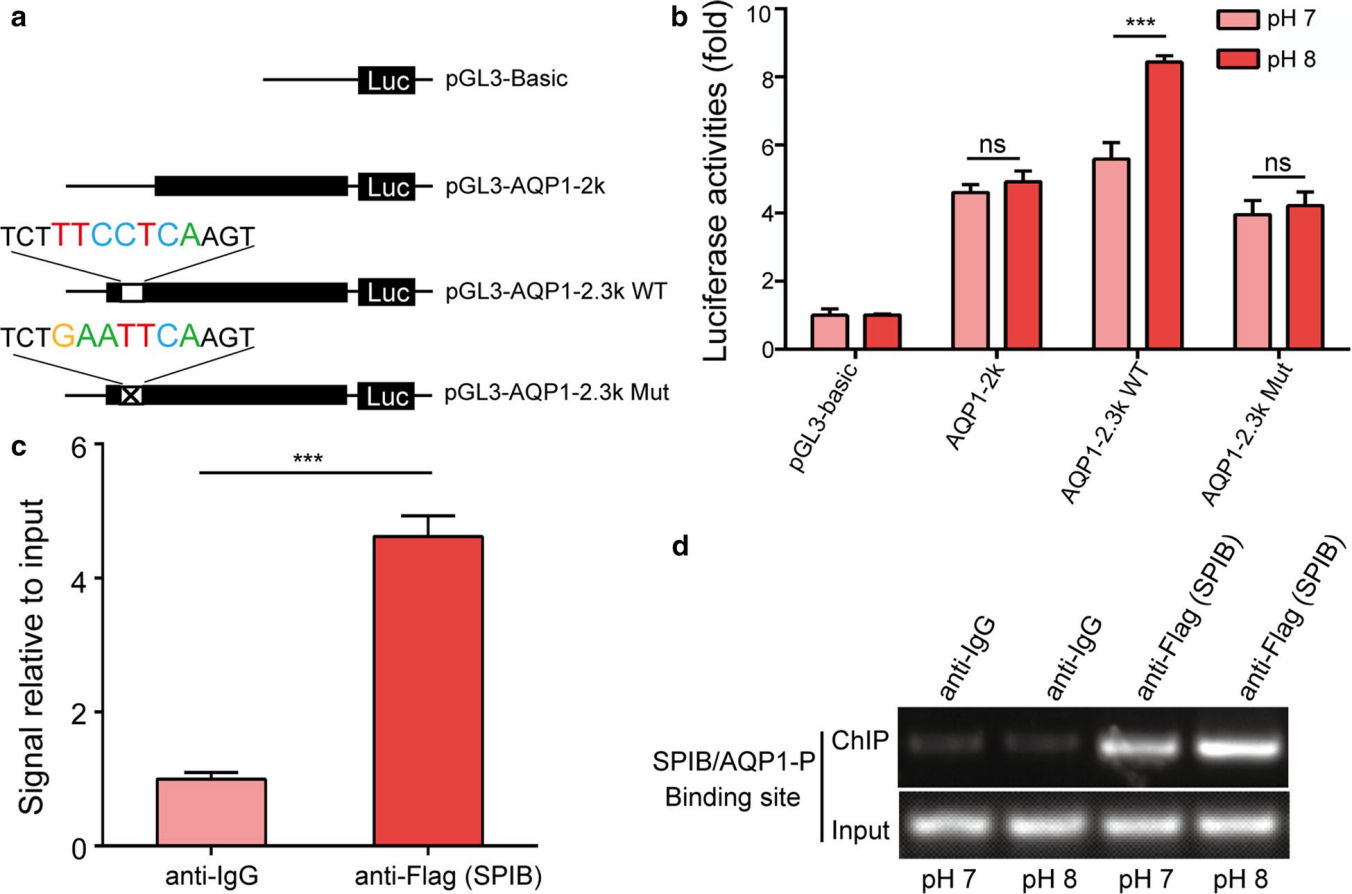
SPIB is a potential transcription factor capable of influencing pH-regulated AQP1 expression. **a** Putative SPIB binding site in the region between − 2300 to − 2200 bp upstream of AQP1. **b** Firefly luciferase activity normalized to Renilla luciferase activity in HEK-293T cells co-transfected with luciferase reporters with the wild-type or mutant AQP1 promoter after treatment at pH 7 or 8. **c** Chromatin immunoprecipitation (ChIP) in HEK293T cells, followed by real-time PCR amplification of the binding site within the AQP1 promoter region. **d** ChIP assays for the binding site in the AQP1 promoter were performed in HEK293T cells under different pH conditions. The data represent the mean ± SEM. *ns* no statistical significance; ***P < 0.001

JASPAR predicted a number of SP1 and THAP1 binding sites in the *AQP1* promoter region between − 1000 and 0 bp. However, no SPIB binding sites were detected. We interfered the expression of the three transcription factors by siRNA and found that the expression of *AQP1* could be inhibited by knockdown of SP1 and THAP1, but not by knockdown of SPIB (Additional file 1: Fig S2). However, the pH-mediated upregulation of *AQP1* mRNA expression was not observed after interference with SPIB gene expression (Fig. 3b). Moreover, a SPIB binding site was predicted in the *AQP1* promoter region between − 2257 and − 2251 bp. Therefore, we hypothesized that SPIB plays a major role in pH-mediated upregulation of *AQP1* expression. To test this hypothesis, luciferase reporter vectors were subjected to site-directed mutagenesis to introduce mutations into the corresponding binding sites located in the *AQP1* promoter region between − 2300 and 0 bp. Subsequently, HEK-293T cells were transfected with the corresponding luciferase reporter vectors (wild-type or mutant, Fig. 4a), cultured and exposed to various pH stimuli. The dual-luciferase activities were then examined in the transfected cells. As shown in Fig. 4b, increased pH resulted in upregulation of luciferase activity from the reporter vector containing wild-type AQP1-2.3k (WT). In contrast, increased pH had no significant effect on the luciferase activity of the reporter vector containing the mutant AQP1-2.3k (MT). The results indicated that the site located in the *AQP1* promoter region between − 2257 and − 2251 bp was activated in a pH-dependent manner. Chromatin immunoprecipitation (ChIP) assays of HEK-293T cells revealed that SPIB could bind to this region (Fig. 4c). Furthermore, an increase in pH caused a dramatic increase in SPIB at the *AQP1* promoter (Fig. 4d), which indicated that SPIB is recruited to the *AQP1* promoter as a DNA-binding complex.

**Fig. 5:**
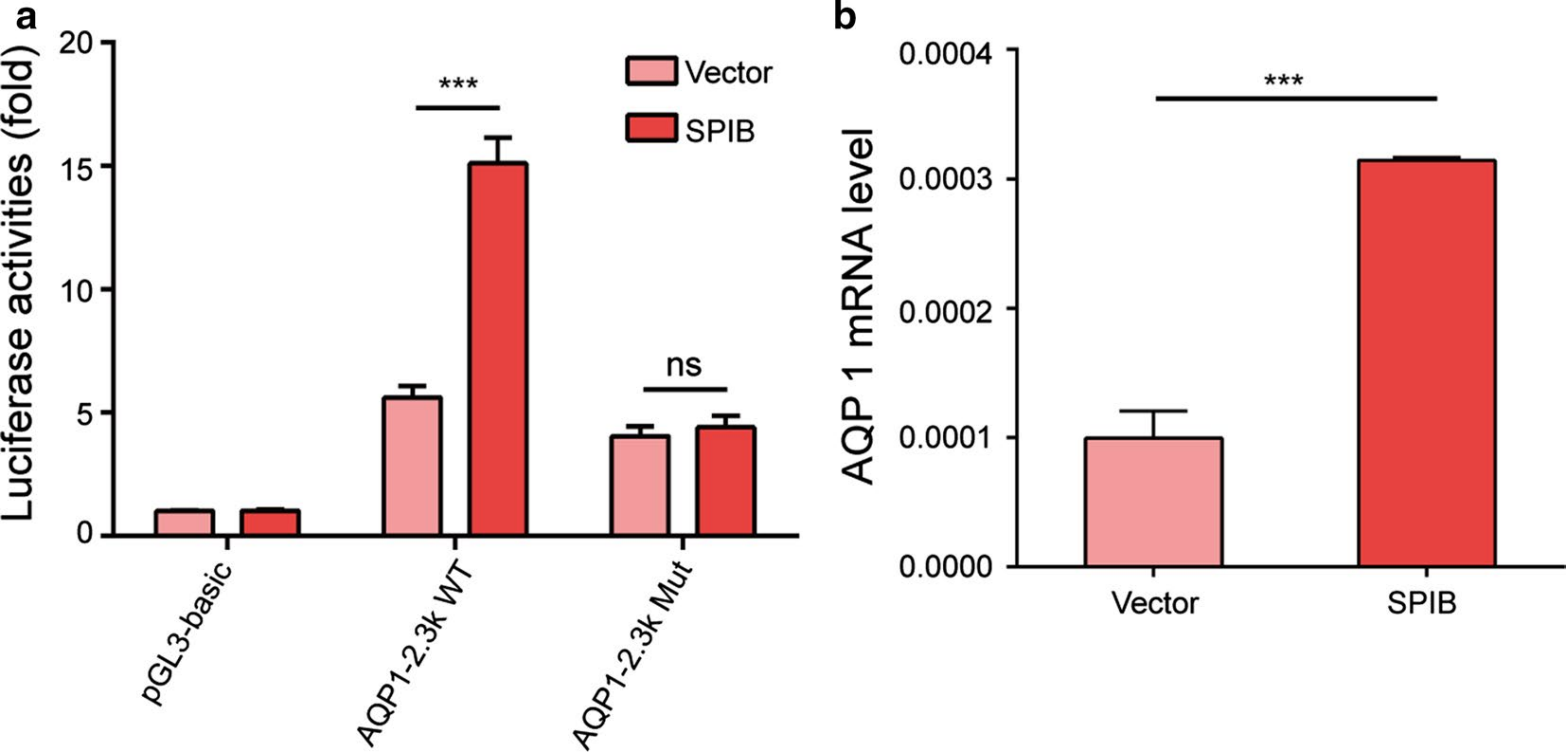
AQP1 is regulated by SPIB. **a** Luciferase activity associated with the region between −2300 and −2200 bp in HEK-293T cells transfected with SPIB. **b** AQP1 mRNA levels in HEK-293T cells transfected with SPIB. The data represent the mean ± SEM. *ns* no statistical significance; ***P < 0.001

We further determined whether SPIB regulates the *AQP1* promoter, which contains a potential SPIB binding site. SPIB overexpression in HEK-293T cells increased the activity of the wild-type *AQP1* promoter but not the mutant *AQP1* promoter and increased *AQP1* mRNA expression (Additional file 1: Fig. S3, Fig. 5). These results demonstrated that SPIB might be a transcription factor capable of affecting the pH-regulated expression of the *AQP1* gene.

## Discussion

Our previous study demonstrated that a new bicarbonate-based PD solution enhances the migratory capacity and damage-repair ability of human peritoneal mesothelial cells by upregulating AQP1, thereby playing an important role in protecting the functional integrity of the peritoneum. AQP1 forms ultra-small pores in the peritoneum, is responsible for transporting water across cell membranes [17, 18] and mediates 50% of peritoneal ultrafiltration activities [14, 19]. Therefore, AQP1 is essential for maintaining PD [15, 20]. In recent years, other important biological effects of AQP1 have also been discovered, including its ability to promote cell migration, wound healing, tumour growth and metastasis and CO_2_ transport across the cell membrane [21–26]. Clearly, a variety of important biological therapeutic effects can be achieved by regulating AQP1. For example, downregulation of AQP1 inhibits tumour blood vessel proliferation and tumour metastasis and reduces cerebral oedema. Increased *AQP1* gene expression improves the ultrafiltration function of the peritoneum [20] enhances the migratory and repair capabilities of peritoneal cells [16] and should therefore maintain the integrity of the peritoneum, thereby prolonging the time until PD failure. Thus, studying *AQP1* gene expression regulation is of great significance.

*AQP1* gene expression is regulated by a variety of factors, including glucocorticoids [27], hydrostatic pressure [12] and hypoxia [28]. The promoter region in the *AQP1* gene contains the glucocorticoid [27] and hypertonicity response element (GRE and HRE, respectively) [29–32], which upregulate AQP1 expression in response to glucocorticoid treatment and increasing hypertonicity and hyperoncoticity. Hypoxia-inducible factor 1-alpha (HIF-1α) binds to the *AQP1* promoter and is involved in hypoxia-induced *AQP1* gene expression [33, 34]. We now provide evidence that in addition to these regulatory mechanisms governing AQP1 gene expression, the *AQP1* promoter contains a pH-sensitive region that upregulates *AQP1* expression in response to increasing pH.

The present study first successfully replicated the findings of our previous study using the HEK-293T cell line as a research tool. *AQP1* mRNA levels were gradually elevated in HEK-293T cells as the pH increased; this behaviour closely resembles the regulatory effects of pH on *AQP1* gene expression in peritoneal mesothelial cells. This result indicates that *AQP1* regulation at the gene transcriptional level is affected by pH. Then, the key transcription factors and their binding sites involved in the pH-mediated regulation of *AQP1* gene expression were explored. The region between − 2300 and − 2200 bp was critical for the pH-induced regulation of *AQP1* gene expression. By combining the above findings with JASPAR predictions and the experimental data obtained from the siRNA-mediated interference of gene expression and mutagenesis of the binding sites, we identified SPIB as a potential transcription factor that affects pH-mediated regulation of *AQP1* gene expression. SPIB binds to the PU-box (5′-GAGGAA-3′) and activate transcription of a reporter plasmid containing PU boxes [35]. SPIB has been reported to be involved in differentiation and maturation of plasmacytoid dendritic cells and to inhibit the differentiation of T, B and natural killer (NK) cells [36, 37]. PD solutions with a physiological pH may increase *AQP1* gene expression and enhance peritoneal ultrafiltration by activating SPIB. Additionally, activated SPIB may participate in inhibition of inflammatory cell activation and reduction of inflammatory stimulation in the peritoneum, thereby achieving long-term protection of peritoneal function.

In the present study, we demonstrated that the pH-mediated upregulation of *AQP1* gene expression was not observed after interference with SPIB gene expression, which indicates that SPIB is involved in pH-mediated regulation of *AQP1* expression. Furthermore, we found that SP1 and THAP1 also affected the luciferase activity induced by pH. However, knockdown of SP1 and THAP1 inhibited the mRNA expression of AQP1, while knockdown of SPIB had no such effect. These results indicated that SPIB may play a major role in the pH-mediated upregulation of AQP1. The regulatory effect of SP1 and THAP1 on the mRNA expression of AQP1 need further investigation. Transcription factors activity are tightly regulated through many ways, such as transcriptional control, post-transcriptional modification, transcriptional co-activators and co-repressors, and interaction with enhancer. Using real-time qPCR and western blot analysis, we showed that different pH levels (pH 7 and pH 8) did not have significant effects on SPIB expression (Additional file 1: Fig S4), which indicates that pH-mediated activation of AQP1 transcription does not occur through the increase of SPIB protein level. Enhancers and their associated factors can regulate expression of genes located far upstream and downstream by looping to the promoters of these genes. The interaction of *AQP1* promoter and SPIB protein may engender specific enhancer-gene interaction and are essential for AQP1 activation mediated by pH. However, additional experiments are needed to better define the role of pH-mediated SPIB activation.

## Conclusions

In summary, the present study demonstrated that a region upstream of the *AQP1* gene (− 2300 to − 2200 bp) contains an enhancer required for pH-mediated regulation of *AQP1* gene expression. SPIB is a specific transcription factor that participates in pH-mediated regulation of *AQP1* gene expression. The present study facilitates further elucidation of the mechanisms by which pH regulates *AQP1* gene expression and the molecular mechanisms by which pH and buffer compounds in PD solutions affect peritoneal membrane integrity and function.

## Additional file

**Additional file 1.** Additional figures and table.
