# Supplementary material for: pH-mediated upregulation of AQP1 gene expression through the Spi-B transcription factor
Source: BMC Mol Biol. 2018 Mar 20;19:4. doi: 10.1186/s12867-018-0104-9 (PMC5859780; doi:10.1186/s12867-018-0104-9)
Supplement: Supplementary file 1 — Additional file 1. Additional figures and table. [file 12867_2018_104_MOESM1_ESM.docx]

**pH-mediated upregulation of *AQP1* gene expression**

**through the Spi-B transcription factor**

Yihui Zhai^1,2^, Hong Xu^1,2,†^, Qian Shen^1,2^, Franz Schaefer^3^, Claus P. Schmitt^3^, Jing Chen^1,2^, Haimei Liu^1,2^, Jialu Liu^1,2^, Jiaojiao Liu^1,2^

^1^Department of Nephrology and Rheumatology, Children's Hospital of Fudan University, Shanghai, China

^2^Shanghai Kidney Development and Pediatric Kidney Disease Research Center, Shanghai, China

^3^Devision of Pediatric Nephrology, Center for Pediatric and Adolescent Medicine, University of Heidelberg, Heidelberg, Germany

**Contact Information**

^†^**Correspondence**: **Hong Xu**, Department of Nephrology and Rheumatology, Children’s Hospital of Fudan University; Shanghai Kidney Development and Pediatric Kidney Disease Research Center, Shanghai, China. Address: No. 399 Wanyuan Road, Shanghai 201102, People’s Republic of China; Email: [hxu@shmu.edu.cn](mailto:hxu@shmu.edu.cn); Tel: +86-21-64931120; Fax: +86-021-64931901.

**Additional Fig. S1**





Figure S1 The knockdown efficiencies of genes measured by q-PCR in HEK 293T cells

The mRNA level in HEK293T cells with knockdown of indicated genes. All results are shown as the mean ± SEM. ***P < 0.001.

**Additional Fig. S2**





Figure S2 the mRNA levels of AQP1 in HEK 293T cells

The AQP1 mRNA level in HEK 293T cells transfected with siRNA against SP1, THAP1 and SPIB. All results are shown as the mean ± SEM. ns, no statistical significance; **P<0.01

**Additional Fig. S3**


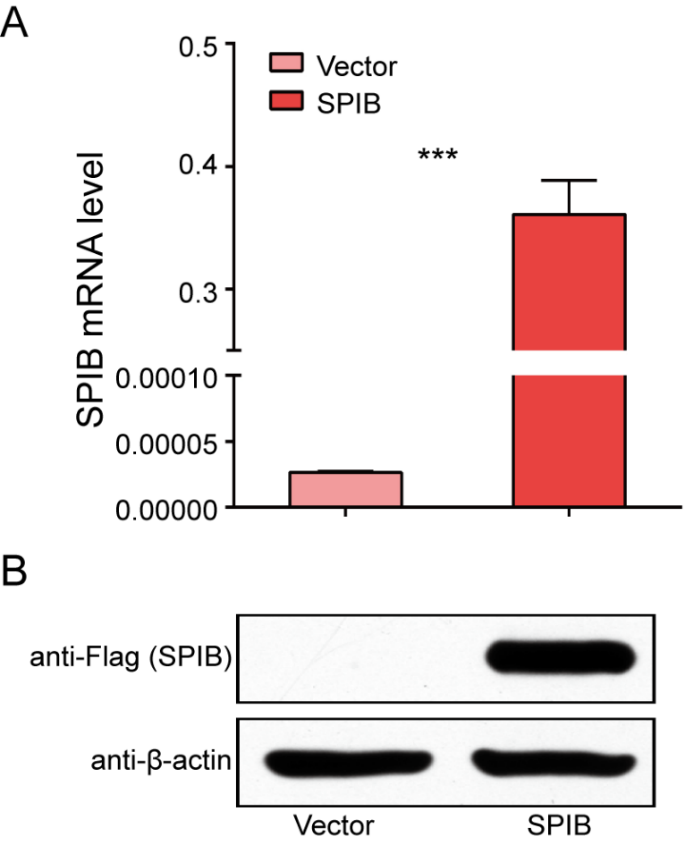


**Figure S3 overexpression of SPIB expression in HEK 293T cells**

1. The SPIB mRNA level in HEK 293T cells transfected with SPIB expression plasmid. All results are shown as the mean ± SEM. ***P < 0.001. (B) the SPIB protein level in HEK 293T cells transfected with SPIB expression plasmid. β-Actin served as a loading control.

**Additional Fig. S4**


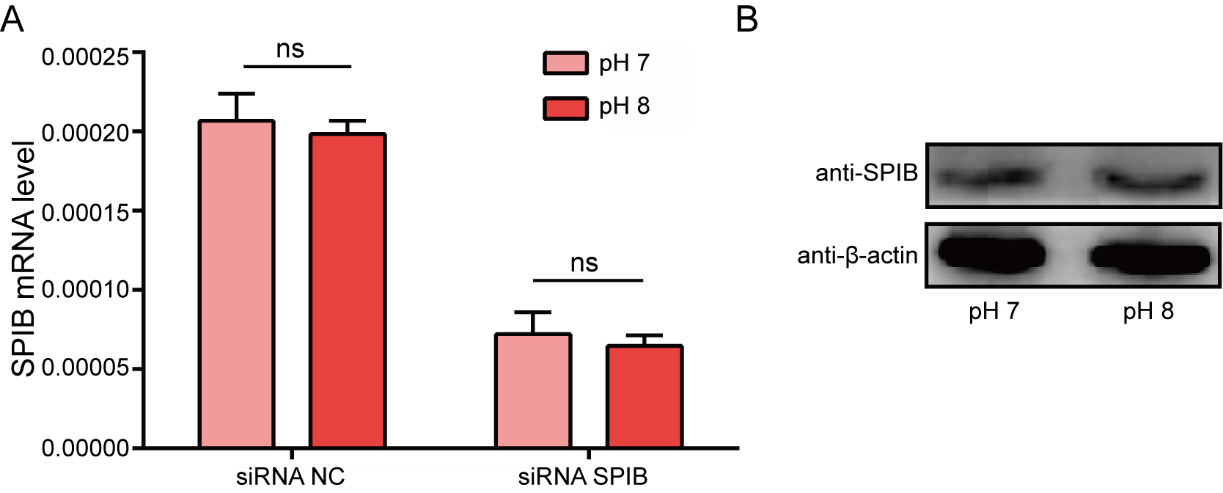


Figure S4 the mRNA and protein levels of SPIB in HEK 293T cells with different pH levels

(A) The SPIB mRNA level in HEK 293T cells transfected with siRNA against SPIB with pH different levels. All results are shown as the mean ± SEM. (B) The SPIB protein level in HEK 293T cells with pH different levels. β-Actin served as a loading control.

**Table S1. Primer sequences**

| Primer name | Sequence (5’to 3’) |
| --- | --- |
| AQP1-q-F | ATGACCTGGCTGATGGTGTGA |
| AQP1-q-R | CGCCTCCGGTCGGTAG |
| SPIB-q-F | CCAGCAGGAACTGGTACAGG |
| SPIB-q-R | AGGTCTCGGACAGCGAGTC |
| EGR1-q-F | GTTTGGCTGGGGTAACTGGT |
| EGR1-q-R | AGCCCTACGAGCACCTGAC |
| FOXL1-q-F | TGATGAACTGGTAGATGCCG |
| FOXL1-q-R | CCTCCCTACAGCTACATCGC |
| KLF5-q-F | TCCCAGGTACACTTGTATGGC |
| KLF5-q-R | ACCCTGGTTGCACAAAAGTT |
| NFIC-q-F | CTTGCTGTCCTCCTGGTCA |
| NFIC-q-R | TGGACCTCTACCTGGCCTAC |
| RFX5-q-F | TGAGGGGAGCTGAAGGTAGA |
| RFX5-q-R | GGCCGTGCAGAACAAAGTAG |
| SP1-q-F | ACCAAGCTGAGCTCCATGAT |
| SP1-q-R | CCTCAGTGCATTGGGTACTTC |
| THAP1-q-F | TTCTTCTGACAGCTGCCTCC |
| THAP1-q-R | ACCGCTACGACAAGGACAAG |
| β-actin-q-F | AGTGTGACGTGGACATCCGCAAAG |
| β-actin-q-R | ATCCACATCTGCTGGAAGGTGGAC |
| AQP1-promoter-F | CCTGAGTTCAGTGGCTCCTTG |
| AQP1-promoter-R | CACCTGTCCCTGCCCTGC TA |
